# Editorial: Women in Cardiac Electrophysiology: 2022

**DOI:** 10.3389/fphys.2023.1299403

**Published:** 2023-10-16

**Authors:** Marianna Meo, Stefanie Fenske, Rasheda Chowdhury

**Affiliations:** ^1^ Boston Scientific Corp, Waltham, MA, United States; ^2^ Center for Drug Research, Department of Pharmacy, Ludwig Maximilian University of Munich, Munich, Germany; ^3^ DZHK (German Centre for Cardiovascular Research), Partner Site Munich Heart Alliance, Munich, Germany; ^4^ National Heart and Lung Institute, Imperial College London, London, United Kingdom

**Keywords:** cardiac electrophysiology, heart rate, ventricle, signaling, monitor, variability, female researcher, collection series

## 1 Introduction

Despite the talent shortage and the increasing demand for a human workforce in the fields of science, technology, engineering, and mathematics (STEM), the role of women in driving scientific progress and developing novel technologies remains marginal, as confirmed by the UNESCO Science Report 2021 (Schmeegans, et al., 2021). Women remain under-represented at the graduate level and the number of women obtaining a doctorate in STEM disciplines remains lower than those earning a baccalaureate in the same specialty (between 19% and 28% (Casad, et al., 2020). The promotion of women to STEM faculty positions is not sufficiently supported and, even in the high-tech industry, despite some effort, the gender gap in technical and leadership roles is still considerable (Schmeegans, et al., 2021), with differences exacerbated by the Coronavirus outbreak. In 2014, among 3810 cardiologists with faculty appointments in the United States, only 16.5% were women (Blumenthal, et al., 2017). In Labinaz, et al. (2019), the analysis of 3396 preclinical studies published in five leading cardiovascular journals over a 10-year period revealed that women accounted for 24% ± 17% of authors per manuscript, with 542 articles (16.0%) not including any female authors and none being authored solely by women.

Cardiac electrophysiology (EP) has one of the lowest representations of women within clinical medicine, due to a variety of factors including a lack of female role models, perceived “old boys’ club” culture, discrimination, and radiation concerns (Michos, 2021). Recent analyses of the Medicare Provider Utilization and Payment Database revealed that on average annually between 2013 and 2019, only 5% of the 3524 EP operators were women. While the total number of EP trainees increased by 75%, the number of women EP trainees decreased by 3% (Howell, et al., 2022). Moreover, one-fifth of the US states had no female EP operator who performed >10 EP procedures of any type as compared with zero states with no male EP operator with at least moderate procedural volume. Despite a 137% increase in atrial fibrillation ablationists between 2013 and 2019, the rate of female operators was only 4% (Howell, et al., 2022).

These data suggest the need for initiatives to encourage women to assume scientific and clinical roles in cardiac EP, managing and performing research projects, and disseminating preclinical and clinical evidence.

## 2 Contributions to the Research Topic

This *Women in Cardiac Electrophysiology 2022* article collection celebrates and promotes the work accomplished by women scientists in the field of cardiac EP. The authors of this editorial are all women, and all the submissions considered were led by women (as the first or last author) demonstrating that the aforementioned gender bias is not due to a lack of capability and expertise among women. A total of six articles have been accepted in this collection, covering a broad range of Research Topic, from basic science to clinical and computational cardiac EP.

The QRS-T angle is a prognostic marker for mortality risk in cardiovascular diseases and can be determined by several methods. The study by Andršová, et al. emphasizes that attention should be paid to the choice of the method used to compute this metric, with the Integral method applied to orthogonalized ECG leads showing the highest intra-subject reproducibility and robustness to data length. QRS-T angle spans a tighter range of values in women, and its relation to heart rate is also gender-specific, suggesting that cardiovascular risk should be addressed differently in distinct patient populations. This study demonstrates that QRS-T angle from the electrocardiogram (ECG) is directly influenced by cardiac autonomic and neurohumoral status and that it is plausible to speculate that spatial QRS-T angle measurement may provide a direct assessment of cardiac autonomic responsiveness at the ventricular level.

While myocardial ischemia is typically associated with action potential duration (APD) shortening due to the activation of IK(ATP) current, the mechanisms underlying the transient prolongation of APD and QTc interval on ECG at the very early stage of ischemia, are poorly understood at the cellular level. The study by Bernikova, et al. confirms in a porcine myocardial ischemia model a repolarization prolongation at an early stage of ischemia that manifests *in vivo* as prolonged QTc and epicardial activation-recovery interval which was most often (but not exclusively) observed at the border and/or ischemic areas. This prolongation was transient and followed by a successive shortening of repolarization. This effect was reproduced in isolated cardiomyocytes by activation of the IK(ATP) current, which led to transient APD prolongation followed by steady APD shortening, with temporal dynamics similar to the *in vivo* situation. Prolonged epicardial activation-recovery interval and surface QTc intervals were both associated with the incidence of phase 1A ventricular fibrillation.

Not only orderly repolarization but also synchrony of intracellular calcium release and t-tubule structure organization in ventricular cardiomyocytes are crucial for maintaining a regular heartbeat and contractile function. Although line scanning confocal microscopy is a standard technology for imaging calcium dynamics in cardiomyocytes, it may show limited spatiotemporal resolution in characterizing calcium dynamics in physiologically anisotropic tissue. A custom light-sheet fluorescence microscope has been designed by Dvinskikh, et al. to perform fast dual-channel 2D timelapse imaging of calcium and the sarcolemma in ventricular cardiomyocytes with low phototoxicity. The use of this system highlighted a correlation between calcium dynamics and t-tubule network coverage of the cell, supporting the idea that disruption of the tubular network would introduce changes in spatial dyssynchrony of calcium dynamics across the cell, affecting the mechanical contractility and uniformity, and hence overall cardiac function.

Abnormal calcium handling in cardiomyocytes is one of the underlying causes of early or delayed afterdepolarizations, which may trigger premature ventricular contractions (PVCs). Treating PVCs is essential to prevent complications associated with ventricular tachycardia such as cardiomyopathy and heart failure. Noninvasive screening through electrocardiographic imaging (ECGi) may allow for preliminary localization of the PVC origin to shorten the duration of invasive radiofrequency catheter ablation thereby reducing procedural times and increasing safety. However, different ECGi formulations and hypotheses may lead to contrasting results, and there is little evidence about the most reliable approach. In Dogrusoz, et al., the authors demonstrate that PVC origin localization is more accurate in dipole-based models than in the potential-based ones. Furthermore, torso inhomogeneities are also shown to affect localization performance, and dipole-based approaches showed satisfactory performance with different torso-heart models. Finally, localization error was higher for spontaneous PVCs than paced, and the potential-based model showed better performance.

Differences between spontaneous and paced PVCs have also been investigated by Bear, et al., who validated ECGi accuracy reconstruction on epicardial recordings from explanted pig hearts in a torso-tank model during hyperkalemia. The authors show that signal morphology and activation map reconstruction are more accurate for spontaneous PVCs than the paced ones, but localization performance was similar. However, spontaneous PVCs were less well reconstructed when the R-on-T phenomenon occurred and the activation wavefronts encountered a line of block, suggesting that the model may be too simplistic when dealing with functional or anatomical obstacles. ECGi reconstruction worsened during K+ perfusion as well, and in particular close to the perfusion site, suggesting again that concomitant pathologies may degrade inverse problem estimation.

Pulsed electric field (PEF) ablation, also known as pulsed-field ablation, has recently emerged as a promising alternative to thermal ablation in the treatment of atrial fibrillation. However, PEF safety and efficacy strongly rely on the characteristics of the PEF waveform, whose parameters require therefore some careful tuning. In response to the still low number of animal and human studies aiming at optimizing PEF waveform, the study by Casciola, et al. puts forward an *in-vitro* model based on human induced pluripotent stem cell-derived cardiomyocytes to investigate the effect of several PEF parameters’ combinations on cell death. Using this model, the authors identified optimal values of PEF threshold duration to guarantee irreversible cell death on a target surface and showed that increasing the number of PEF pulses beyond the experimentally determined threshold only contributes to tissue heating with no increased benefit for therapy efficacy by modeling the temperature field distribution.

## 3 Conclusion

This Research Topic demonstrates the strength and quality of meaningful scientific, transversal contributions from female scientists, addressing some important questions in the field of cardiac EP using diverse approaches. We hope this encourages other women researchers to follow a similar path and share their work with the EP community.
